# Supplementary material for: Integrating emotional intelligence, memory architecture, and gestures to achieve empathetic humanoid robot interaction in an educational setting
Source: Front Robot AI. 2025 Sep 4;12:1635419. doi: 10.3389/frobt.2025.1635419 (PMC12444663; doi:10.3389/frobt.2025.1635419)
Supplement: Supplementary file 1 [file Supplementaryfile1.pdf]

A. Appendix: Course App Interface

Supplementary Figure 1 illustrates the user interface of the Course App used during the robot tutoring session.

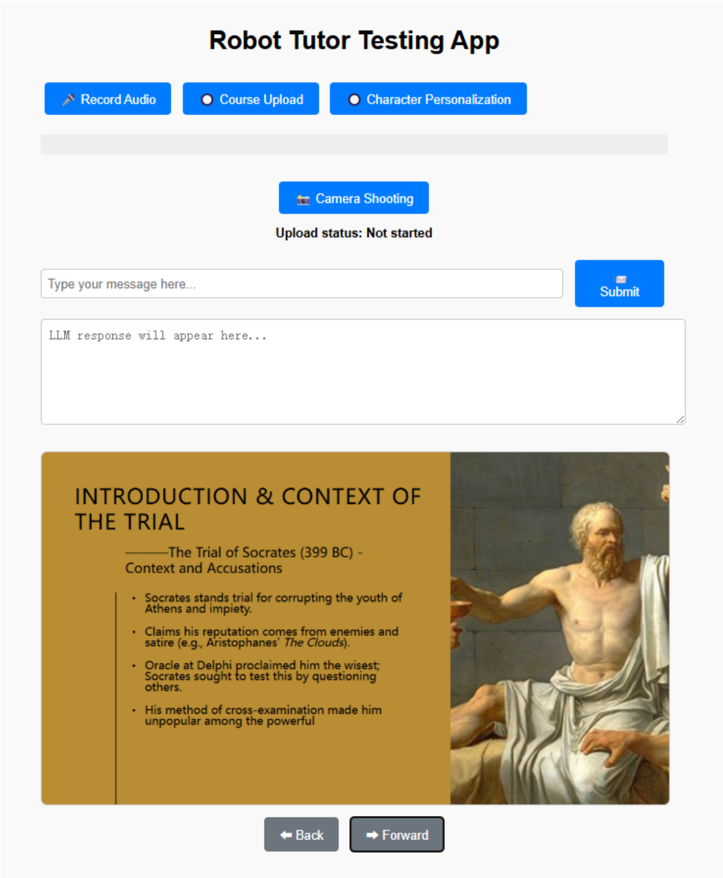

Supplementary Figure 1. User interface of the Robot Tutor Course App. The **Record Audio** button enables the robot to start listening to the student's speech in real time. The **Course Upload** button allows scaling the robot tutor to cover multiple subjects. The **Character Personalization** button adapts the robot's persona to suit individual student needs. The **Camera Shooting** button activates facial expression capture for inferring the student's emotional state. The text input box provides an alternative input method when speech recognition fails, due to strong accents. The **LLM response** section displays the robot's textual reply as a complement to its spoken response, ensuring clarity for the student. The course slide at the bottom illustrates a history lesson on the Trial of Socrates, demonstrating the system's educational content delivery.

B. Appendix: Post-Experiment Questionnaire

Section 1: Engagement
Q1. The robot tutor maintained my attention throughout the session. ( )
Q2. The robot's gestures or expressions helped me stay engaged with the content. ( )

Scale: 1 = Strongly Disagree to 5 = Strongly Agree

Section 2: Satisfaction
Q3. I enjoyed learning with the robot tutor. ( )
Q4. I would be interested in using this kind of robot tutor in other classes. ( )

Scale: 1 = Strongly Disagree to 5 = Strongly Agree

Section 3: Perceived Effectiveness
Q5. The robot adapted its behavior based on my responses or emotional state. ( )
Q6. I understood the topic better after the session with the robot tutor. ( )

Scale: 1 = Strongly Disagree to 5 = Strongly Agree

Section 4: Open-Ended Feedback
Q7. What was the most effective or memorable aspect of the robot tutor?

Q8. What would you change or improve about the robot's behavior or teaching style?
